# A Novel Assessment Model Based on Molecular Subtypes of Hypoxia-Related LncRNAs for Prognosis of Bladder Cancer

**DOI:** 10.3389/fcell.2021.718991

**Published:** 2021-11-15

**Authors:** Xianwu Chen, Yan Zhang, Feifan Wang, Xuejian Zhou, Qinghe Fu, Xintao Yang, Juntao Lin, Xiaodong Jin

**Affiliations:** Department of Urology, The First Affiliated Hospital, Zhejiang University School of Medicine, Hangzhou, China

**Keywords:** bladder cancer, hypoxia, lncRNA, molecular subtype, prognostic risk model

## Abstract

Hypoxia is a common feature in various tumors that regulates aggressiveness. Previous studies have demonstrated that some dysregulated long non-coding RNAs (lncRNAs) are correlated with tumor progression, including bladder cancer (BCa). However, the prognostic effect of hypoxia-related lncRNAs (HRLs) and their clinical relevance, as well as their regulatory effect on the tumor immune microenvironment, are largely unknown in BCa. A co-expression analysis between hypoxia genes and lncRNA expression, which was downloaded from the TCGA database, was performed to identify HRLs. Univariate Cox regression analysis was performed to select the most desirable lncRNAs for molecular subtype, and further LASSO analysis was performed to develop a prognostic model. This molecular subtype based on four HRLs (AC104653, AL136084, AL139393, and LINC00892) showed good performance in the tumor microenvironment and tumor mutation burden. The prognostic risk model suggested better performance in predicting BCa patients’ prognosis and obtained a close correlation with clinicopathologic features. Furthermore, four of five first-line clinical chemotherapies showed different sensitivities to this model, and nine immune checkpoints showed different expression in the molecular subtypes or the risk model. In conclusion, this study indicates that this molecular subtype and risk model based on HRLs may be useful in improving the prognostic prediction of BCa patients with different clinical situations and may help to find a useful target for tumor therapy.

## Introduction

More than 570,000 patients were diagnosed with bladder cancer (BCa) in the past year, making BCa one of the top 10 cancers with high morbidity and mortality in the world, which is a common malignant tumor of the urinary system with a poor prognosis (Sung et al., 2021). In current clinical practice, pathological biopsy with cystoscopy is considered to be the most reliable method for detecting this highly heterogeneous cancer (Cumberbatch and Noon, 2019). In all BCa cases, more than 75% of cases are non-muscle invasive (NMIBC), and the primary and traditional treatment option mainly includes surgery (transurethral resection of bladder tumors, TURBT) with or without chemotherapy (intravesical therapy, IVT) (Bryan et al., 2021). However, almost a quarter of NMIBC patients ultimately develop muscle-invasive BCa (MIBC) because of its high recurrence rate and the occurrence of chemical resistance (Babjuk et al., 2019). In recent years, although immunotherapies using immune checkpoint inhibitors have gradually been used as a treatment, there are no biomarkers to assess the effectiveness of immunotherapy in BCa. Therefore, novel and accurate therapeutic evaluations for BCa treatment are urgently needed.

Cellular hypoxia is a common feature in various physiological or pathophysiological states in the tumor microenvironment (TME) and plays critical roles in cancer initiation, progression, and treatment resistance in various cancers, including BCa (Reiher et al., 2001; Wan et al., 2014). With the development of gene-sequencing technology, many hypoxia-related signatures have been established in multiple tumors to predict patient prognosis and treatment efficacy, such as immunotherapy and chemotherapy response (Shao et al., 2021; Sun et al., 2021a). In BCa, multiple hypoxia-related gene signatures were developed and validated to predict prognosis and TME immune characteristics (Yang et al., 2017; Liu et al., 2021). In recent years, with the successful application of immunotherapy in tumors, an increasing number of people have begun to pay attention to the clinical value of immune checkpoints, such as anti-PD-L1/PD-1 therapy. It is worth noting that the effect of immunotherapy is closely related to the TME (Hu et al., 2021; Liu et al., 2021). Liu et al. (2021) developed and validated a hypoxia risk score that was related to the expression of several immune checkpoints, such as PD-L1, PD-1, CTLA-4, and LAG-3, by analyzing the BCa TME.

Long non-coding RNAs are untranslated RNAs of more than 200 nucleotides in length, and over 15,000 lncRNAs are encoded by the human genome (Zhang et al., 2017). Dysregulated lncRNA expression is correlated with cancer progression and has been reported in different cancer types, including BCa (Liang et al., 2018). Several hypoxia-related lncRNAs (HRLs) have been certified to play crucial roles in tumor regulation or processes (Yuan et al., 2019). For instance, hypoxic cells remodel the tumor microenvironment to facilitate tumor growth and development by secreting oncogenic lncRNA UCA1 enriched exosomes (Xue et al., 2017). LncRNA HOXA-AS3 could act as a competing endogenous RNA (ceRNA) of miR-455-5p to regulate Notch1 and regulate chemotherapeutic drug sensitivity in BCa cells (Chen et al., 2020).

However, only a few studies have reported the role of several HRLs in BCa without its clinical prognostic value and its impact in the TME. Therefore, understanding HRL differential expression in BCa patients may help to identify biomarkers that can act as useful therapeutic targets and predict patient prognosis.

The present study intends to classify the expression of HRLs in BCa patients and develop a model with bioinformatics methods to investigate the influence of the TME, and predict patient prognosis, chemotherapy, and immunotherapy response.

## Materials and Methods

### Data Source

The expression of RNAseq (including mRNAs and lncRNAs) and clinical information (including age, gender, tumor grade, disease stage, TNM stage, and prognosis) were obtained from The Cancer Genome Atlas database (TCGA^1^) by using the keywords “TCGA-BLCA AND HTSeq-FPKM AND Gene Expression Quantification” on April 4, 2021. Patients with incomplete information were excluded.

The hypoxia genes were searched from the Molecular Signatures Database v7.4 of GSEA^2^. The gene was excluded if gene expression was not detected in more than half of the BCa patient samples.

### Correlation Analysis

To identify the HRLs, Pearson correlation analysis was performed between different expression levels of hypoxia genes (log_2_FC of > 1 and FDR of < 0.05) and all lncRNA expression data in samples to identify the HRLs (| correlation coefficient| ≥ 0.4 and *p* < 0.001). The heatmap of correlation analysis was performed using the OmicStudio tools at https://www.omicstudio.cn/tool.

### Cell Lines and RT-qPCR Analysis

A normal human bladder epithelial cell line (SV-HUC1) and BCa cell lines (T24, TCCSUP, and UMUC-3) were obtained from the American Type Culture Collection (ATCC; Manassas, VA, United States). The cells were cultured in RPMI 1640 medium (Hyclone), supplemented with 10% fetal bovine serum (Gibco) and 1% penicillin/streptomycin, in a humidified incubator (Thermo) at 37°C with 5% CO_2_. Total RNAs were extracted from the collection cells using Trizol Kit (Invitrogen, Carlsbad, CA, United States) according to the manufacturer’s instructions. The first strand (cDNA) was reverse-transcribed and used as the template for qPCR analysis as previously described (Wang et al., 2020). The expression levels of lncRNAs were measured using the SYBR Green qPCR Kit (Takara, Japan). The gene name and primer sequences were listed in Supplementary Table 1. Glyceraldehyde 3-phosphate dehydrogenase (GAPDH) was used as an endogenous control.

### Consensus Clustering

Univariate Cox regression analysis was used to extract the HRLs *via* the R package “survival” (*p*-value ≤ 0.05). Consensus clustering was performed to explore the potential molecular subtype between the BCa patients using the R package “ConsensusClusterPlus.” The count of cluster (*k*) was set from 1 to 9, and the best optimal *k* value was selected for further investigation.

### Gene Set Enrichment Analysis (GSEA)

Gene set enrichment analysis (version 4.1.0) was performed to identify the differences in the set of genes expressed between the two clusters in the enrichment of the Kyoto Encyclopedia of Genes and Genomes (KEGG) pathway. The number of permutations was performed 1,000 times for each analysis, and the pathways with FDR < 0.05 were considered statistically significant.

### Estimation of Tumor Immune Microenvironment

An analytical tool, CIBERSORT, was used to evaluate the tumor immune infiltration levels of BCa by providing an estimation of the abundances of 22 kinds of cell types based on all gene expression levels. The algorithm was run for 1,000 permutations and BCa samples with an output *p* < 0.05 were selected for further analysis. The immune score, stromal score, and ESTIMATE score were calculated by the R package “ESTIMATE.”

### Estimation of Tumor Mutational Burden (TMB)

Tumor mutational burden is an emerging therapeutic measure of sensitivity to immunotherapy. The R package “maftools” was used to process somatic mutation data including somatic, coding, base replacement, and insert–deletion mutations. The median TMB value was defined as the cutoff value to separate BCa patients into High-TMB and Low-TMB groups.

### Development of the Hypoxia-Related LncRNAs Prognostic Model

All BCa samples were randomly equally divided into construction and verification datasets at a ratio of 1:1. A least absolute shrinkage and selection operator (LASSO) Cox hazards model analysis was used to construct the HRL risk model with the optimal prognostic value by the R package “glmnet.” The following formula was used to calculate the risk score of every patient according to the regression coefficients from the model and lncRNAs expression: $Riskscore=\sum_{i}^{n}\left(coef\times expression\right)$ (*n*: the number of lncRNAs; *i*: the serial number of each lncRNA; *coef*: the coefficient value from the Cox hazards analysis; *expression*: HRL expression). Univariate and multivariate analyses were performed to verify the independent prognostic predictors *via* the R package “survival.” A nomogram was used to predict the 1-, 3-, and 5-year survival ratio based on the clinicopathologic features (such as age, gender, tumor grade, and disease stage) and risk score of this model to assist clinical procedures and improve risk stratification by using the R package “rms.”

### Predicting Chemotherapeutic Response

Based on the information retrieved from the Genomics of Drug Sensitivity in Cancer (GDSC) database^3^, the chemotherapeutic sensitivity for each BCa was calculated. Five common clinical chemotherapeutic drugs were selected to predict the chemotherapeutic response, and the R package “pRRophetic” was used to estimate the chemotherapeutic response determined by the half-maximal inhibitory concentration (IC50).

### Statistical Analysis

R software (version 4.0.3), and GraphPad Prism (version 6.0) were used to analyze the statistical significance. One-way ANOVA followed by *ad hoc* Tukey’s multiple comparisons was utilized to compare the expression of lncRNAs in different cell lines. The Wilcoxon signed-rank test was used to analyze continuous variables, whereas the chi-square test was used to analyze categorical data. Kaplan–Meier curves were plotted, and a log-rank test was used to calculate the significant survival difference. The sensitivity and specificity of prognostic prediction using the HRL risk score were analyzed by Receiver Operating Characteristic (ROC) analysis. The area under the ROC curve (AUC) served as an indicator to validate prognostic accuracy. A *p* < 0.05 was considered statistically significant.

## Results

### Identification of Hypoxia-Related LncRNAs

**FIGURE 1:**
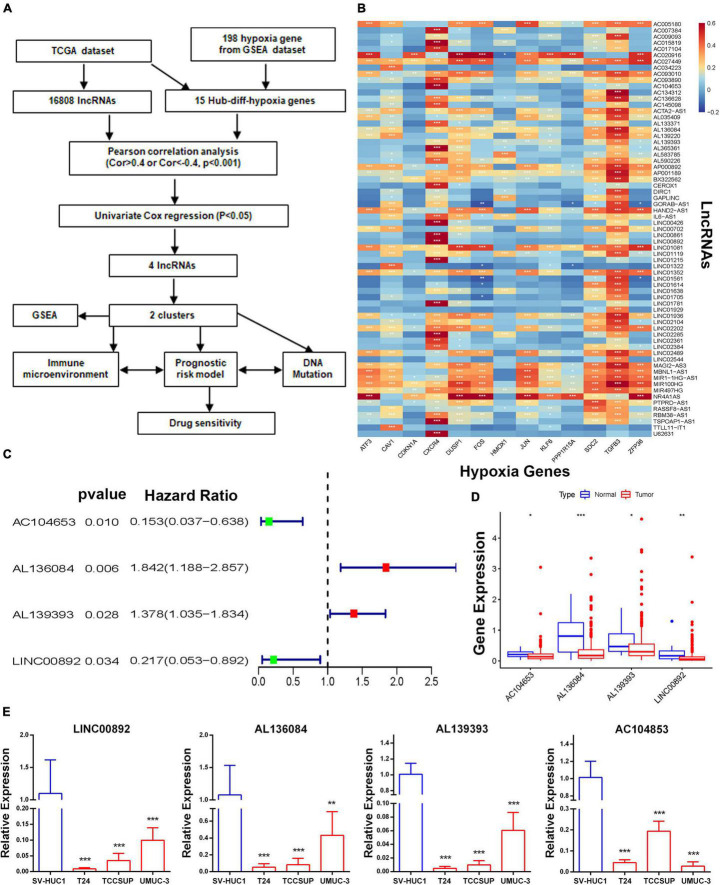
Identification of a molecular subtype of HRLs. **(A)** Study flow chart. **(B)** The heatmap of the correlations between hypoxia-related genes and hypoxia-related lncRNAs. **(C)** The four hypoxia-related prognostic lncRNAs. **(D)** The expression of four lncRNAs. **(E)** The expression level of four HRLs in a normal bladder cell line (SV-HUC1) and tumor cell lines (T24, TCCSUP, and UMUC-3), *p < 0.05, **p < 0.01 and ***p < 0.001.

A total of 16,808 lncRNAs and 20,454 mRNAs from 414 BCa tumor samples and 19 non-tumor samples were downloaded from the TCGA database. Of the 198 hypoxia genes selected from the GSEA database, 65 differentially expressed hypoxia genes were identified in the TCGA-BLCA cohort (Supplementary Figures 1A,B). Among them, the expression matrixes of 15 hub hypoxia-related genes were extracted. In total, by co-expression analyses, 67 HRLs were confirmed (Supplementary Figures 1C,D). Combined with patient prognostic information downloaded from TCGA datasets, univariate Cox regression was then implemented to screen HRLs from these 67 genes (Figures 1A,B), and four HRLs (AC104653, AL136084, AL139393, and LINC00892) were significantly related to BCa patient prognosis and significantly differentially expressed in tumor and non-tumor specimens (Figures 1C,D). Correspondingly, RT-qPCR analysis of basic experiments also demonstrated that the expression level of four HRLs was lower in tumor cell lines (T24, TCCSUP, and UMUC-3) than in a normal bladder cell line (SV-HUC1) (Figure 1E).

### The Molecular Subtype Based on the Hypoxia-Related LncRNAs

**FIGURE 2:**
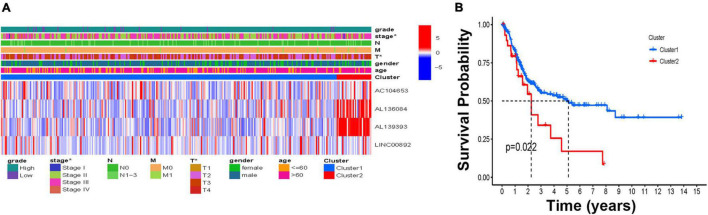
The molecular subtype based on the HRLs. **(A)** The heatmap of the correlations of molecular subtype and clinical features. **(B)** Kaplan–Meier curve of two molecular subtypes, *p < 0.05, **p < 0.01, and ***p < 0.001.

To investigate the connections between the expression of these four HRLs and BCa subtypes, consensus clustering analysis was performed with BCa patients. The results showed that when *k* = 2, patients are divided into two clusters with the highest intragroup correlations and the lowest intergroup correlations (Supplementary Figure 2). A relationship between patient clusters and the gene expression profiles and their clinical features, such as grade, stage, TNM stage, gender, and age, are shown in a heatmap, indicating that there is a significant difference in disease stage between the two clusters (Figure 2A). Additionally, the Kaplan–Meier curve showed that patients in Cluster 1 had a significantly prolonged overall survival (OS) ratio compared with those in Cluster 2 (Figure 2B).

### Gene Set Enrichment Analysis Enrichment Analysis of Molecular Subtypes

To investigate which pathways were enriched in clusters, GSEA was performed to identify the significant KEGG pathways associated with the two clusters in BCa patients. Patients in Cluster 2 were mainly enriched in the MAPK signaling pathway, TGFβ signaling pathway, angiotensin system, ligand receptor interaction, smooth muscle contraction, calcium signaling pathway, leukocyte transendothelial migration, and focal adhesion. However, patients in Cluster 1 were enriched in oxidative phosphorylation (FDR < 0.05) (Supplementary Figure 3).

### Immune Function Analysis of the Molecular Subtypes

**FIGURE 3:**
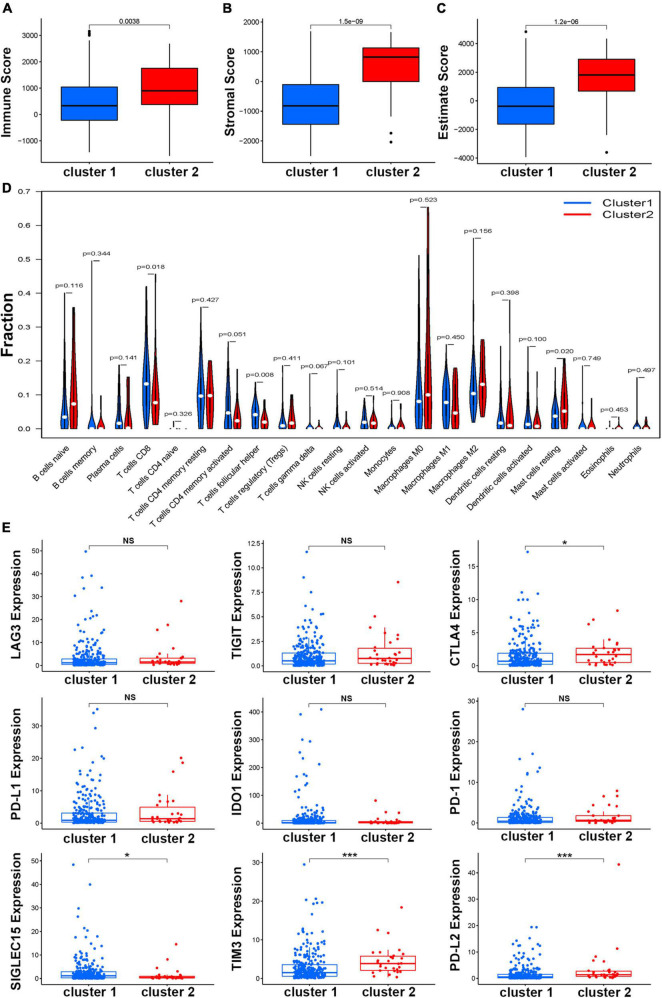
Estimation of the function of the immune cells. The boxplot of correlation between molecular subtypes and immune score **(A)**, stromal score **(B)**, and estimate score **(C)**. **(D)** Relationships between the molecular subtype and the immune cell infiltration in BCa. **(E)** The expression of nine immune checkpoints in different molecular subtypes, *p < 0.05, **p < 0.01, and ***p < 0.001.

To explore the difference in immune function in the two clusters, the ESTIMATE score, immune score, and stromal score of BCa samples were calculated. The results showed that patients in Cluster 2 had a significantly higher score than those in Cluster 1 in three kinds of scores (Figures 3A–C). Further investigation of the specific distribution of immune cell fractions in BCa indicated that the Cluster 1 group contained a higher proportion of follicular helper T cells while the Cluster 2 group contained a higher proportion of T cells CD8 and mast cells resting (Figure 3D). Meanwhile, more than 80 immune genes were differentially expressed in these two clusters, indicating that HRL molecular subtypes are associated with immune genes and the tumor microenvironment (Supplementary Figure 4). On the other hand, in recent years, with the advancement of the concept of precision medicine, immune checkpoint inhibitor (ICI) therapy has attracted great attention. Therefore, the different expression levels of ICIs in different subtypes were further explored. Among nine known immune checkpoints (CTLA4, PD-L1, PD-L2, PD-1, SIGLEC15, TIM3, TIGIT, IDO1, and LAG3), there was no difference between normal and tumor samples (Supplementary Figure 5). Remarkably, the overall expression levels of genes including CTLA4, TIM3, and PD-L2 were relatively higher in Cluster 2 while SIGLEC15 was higher in Cluster 1, and the other five genes showed no difference in expression (Figure 3E).

### Tumor Mutational Burden Analysis of the Molecular Subtypes

**FIGURE 4:**
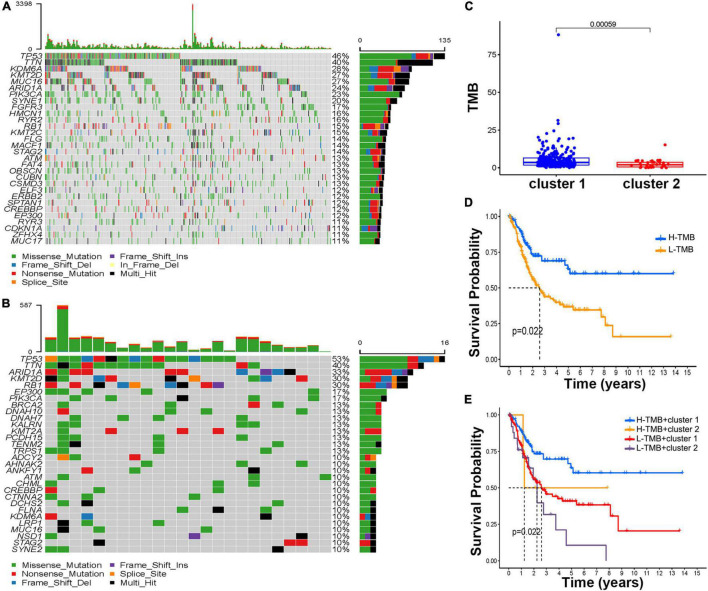
Estimation of the tumor mutation burden (TMB). The waterfall plot of mutation genes in cluster 1 **(A)** and cluster 2 **(B)**. **(C)** The boxplot of the TMB score in the different molecular subtypes. **(D)** Kaplan–Meier curve of H-TMB and L-TMB. **(E)** Kaplan–Meier curve of different TMB scores and molecular subtypes.

After BCa patients were divided into H-TMB (*n* = 113) and L-TMB (*n* = 209) by the median value of the TMB score, the waterfall plots revealed the top mutated genes in the two clusters (Figures 4A,B). Overall, comparing the most frequent somatic mutations between the two clusters, TP53 and TTN were similar between the two clusters in terms of their mutation frequencies. However, the mutation frequencies of ARID1A, KMT2D, and RB1 were ranked third, fourth, and fifth in Cluster 2 (33, 30, and 30%, respectively) while the mutation frequencies were lower than 30% in Cluster 1. The Kaplan–Meier results indicated that H-TMB can prolong patient survival compared with the L-TMB (Figure 4C). Further investigation in cluster subtypes showed that patients in Cluster 2 had a lower TMB score than those in Cluster 1 (Figure 4D). Interestingly, in a study on whether patients in different clusters with different TMB scores would have different prognoses, the Kaplan–Meier analysis demonstrated that patients in Cluster 1 with H-TMB had a significantly better prognosis than patients in other subtypes (Figure 4E).

### Development of a Prognostic Risk Model

**FIGURE 5:**
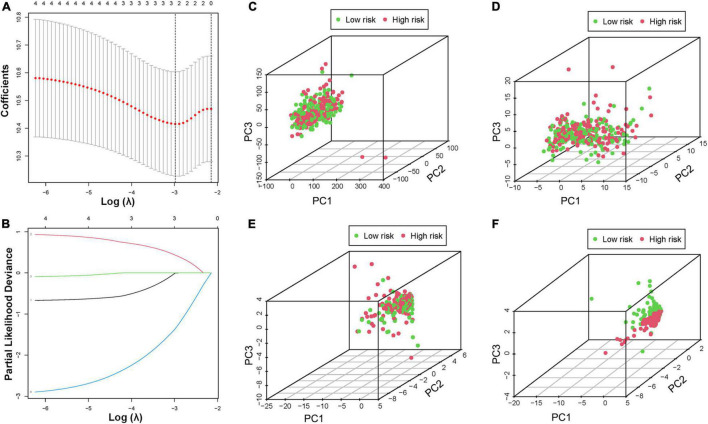
Construction of the prognostic risk model. **(A,B)** LASSO regression of the minimum criteria calculation. PCA of risk score based on the expression profiles of all genes **(C)**, hypoxia genes **(D)**, all HRLs **(E)**, and HRL prognostic risk-related genes **(F)**.

Bladder cancer patients who matched their clinical features and survival information were selected from all BCa samples. Based on four tumor classification lncRNAs, three optimal lncRNAs (AC104653, AL136084, and LINC00892) were selected to construct a model due to the coefficient caused by the LASSO Cox regression analysis (Figures 5A,B). The risk score was calculated according to the following formula: risk score = (−0.005 × AC104653 expression) + (0.431 × AL136084 expression) + (−1.361 × LINC00892 expression). According to this model, all BCa patients were divided into two risk groups with the median score as the cutoff. Principal component analysis (PCA) was used to further verify the risk model, and the results confirmed complete separation between different risk sample sets in the PCA distribution 3D plot (Figures 5C–F). In addition, the Kaplan–Meier analysis of patient prognosis showed that patients in the low-risk group had a higher OS ratio than those in the high-risk group (Supplementary Figure 6).

### Validation of the Risk Model

**FIGURE 6:**
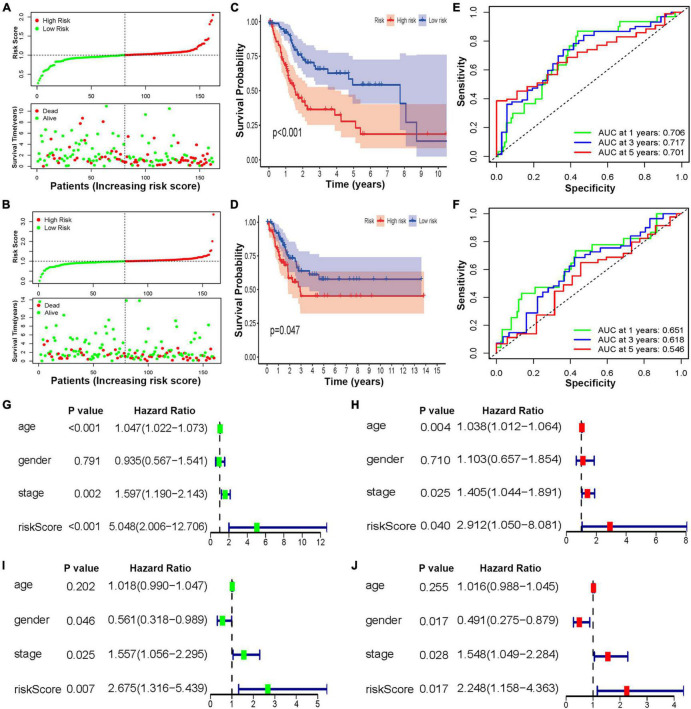
The construction and verification of the HRL risk model of BCa patients. Distribution of the three lncRNA-based risk scores and vital statuses of patients in different risk groups in the construction **(A)** and verification cohort **(B)**. Kaplan–Meier survival curves of OS of different risk patients in construction **(C)** and verification cohort **(D)**. ROC curve of the prognostic model in the construction **(E)** and verification cohort **(F)**. **(G,H)** The univariate and multivariate analyses of patients in the construction cohort. **(I,J)** The univariate and multivariate analyses of patients in the verification cohort.

To reveal the accuracy of the risk score, BCa samples were randomly divided into two cohorts for construction and verification. In both sets of cohorts, the distribution of the risk scores and vital statuses of patients, sorted by the risk score, was the same (Figures 6A,B). The Kaplan–Meier curve results also suggested that the OS ratio of high-risk patients was significantly lower than that of the low-risk group (Figures 6C,D). Moreover, time-dependent ROC analyses also found that this model was an effective way to predict BCa patient prognosis (Figures 6E,F). Univariate and multivariate analyses were used to further investigate BCa patients’ independent prognostic factors and indicated that the risk score was an independent prognostic factor in the construction cohort, considering patient age, gender, and clinical stage as in the verification cohort (Figures 6G–J). Based on the ROC analysis results generated by the risk model, the AUC value was 0.68, which was higher than that of clinicopathologic features, including age, gender, grade, and disease stage, indicating that the prognostic model has a better ability to predict the survival of patients (Supplementary Figure 7).

### Stratification Analysis of the Risk Score Clinical Utility

**FIGURE 7:**
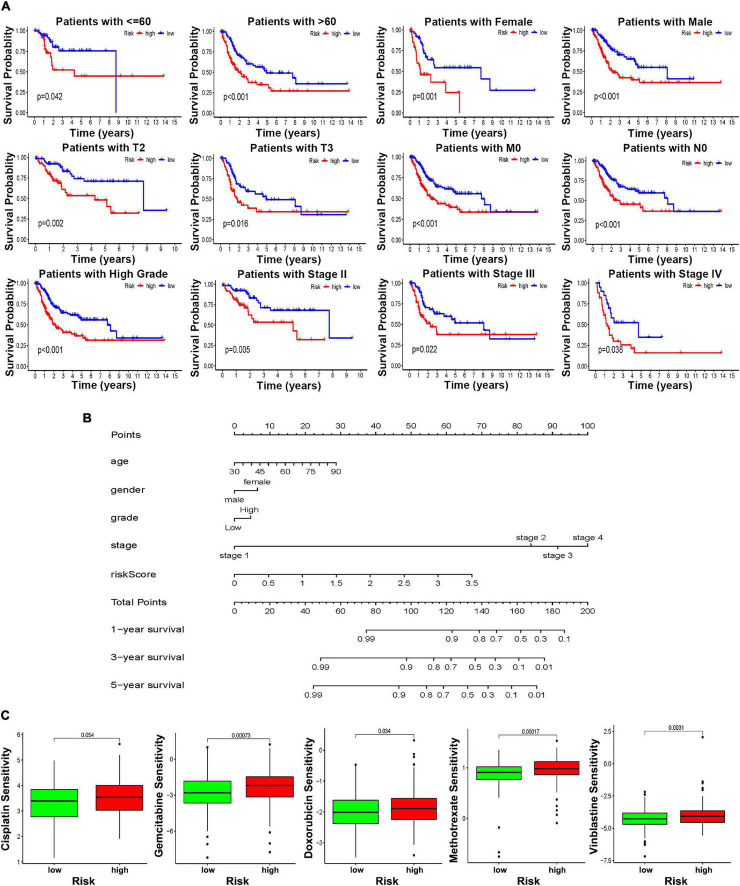
The clinical utility of the risk score. **(A)** Kaplan–Meier survival curves of OS of high and low risk in different clinical stratification. **(B)** Prognostic survival is predicted in the nomographic chart. **(C)** Different chemotherapeutic responses in different risk BCa patients.

To further explore the clinical value of this risk score with different clinical features, BCa patients were separated into different groups based on age, gender, grade, and disease stage. Considering the different stratified analysis results, the BCa patients with high-risk scores predict significantly poorer OS than those with low-risk scores aged ≤ 60 or > 60. Likewise, the results were confirmed in females or males, with high tumor stage (T1–T3), with high disease stage (stage II–IV), with high tumor grade, and without lymph node or distant metastasis (N0 and M0) (Figure 7A). Of note, the results to identify whether clinicopathological features were associated with the risk score revealed a significantly increasing trend in age, T stage, and N stage (Supplementary Figure 8). Referring to all the above findings, based on the risk model score and clinicopathological features, the nomogram was established as a clinically applicable quantitative tool to predict the OS of BCa patients (Figure 7B). In addition, considering the clinical treatment value, the high-risk patients showed more sensitivity to gemcitabine, doxorubicin, methotrexate, and vinblastine than the low-risk group, while there was no significant difference in sensitivity to cisplatin (Figure 7C).

### Associations Between the Risk Model and Molecular Subtype and Immune Function

**FIGURE 8:**
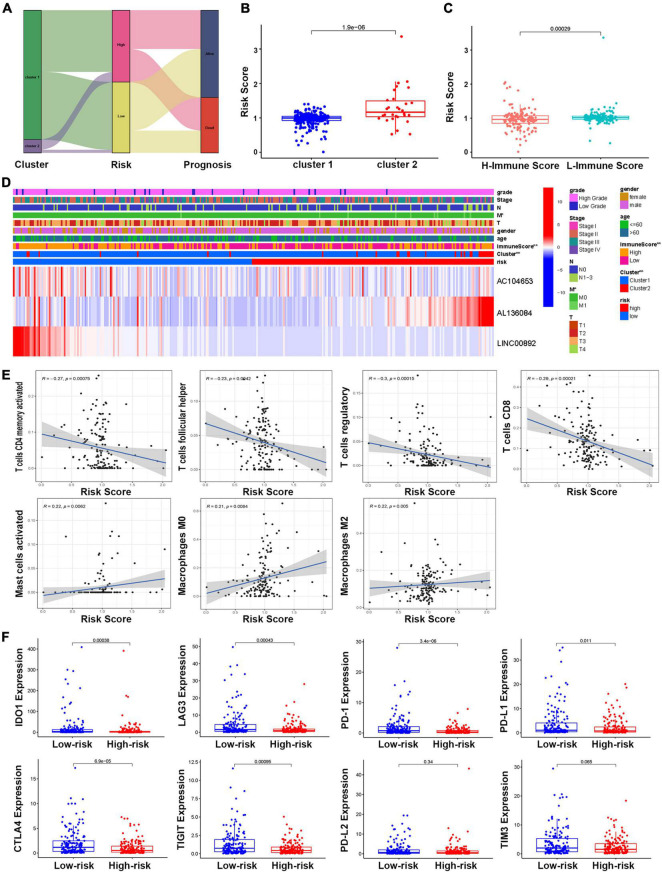
The relationship between the risk model and molecular subtype and immune function. **(A)** The Sankey diagram of the relationship between molecular subtype, risk score, and prognosis. **(B)** The boxplot of the relationship between risk score and molecular subtypes. **(C)** The boxplot of the relationship between risk score and immune score groups. **(D)** The heatmap of the correlations of risk score with molecular subtype and clinical features. **(E)** Correlations between risk score and immune infiltrating level of seven types of immune cells. **(F)** Comparison of the expression of selected immune checkpoint in high- and low-risk groups.

A Sankey diagram was established to illustrate the relationship of molecular subtype and risk score and patient prognosis. The results showed that more than half of the patients in Cluster 2 could be classified as high-risk and predict poor survival (Figure 8A). Moreover, the boxplot results also supported that BCa patients’ risk score in Cluster 2 was significantly higher than that of patients in Cluster 1 (Figure 8B). For the correlation between immune score and risk score, the boxplot showed that a high immune score was negatively correlated with a risk score (Figure 8C). The heatmap analysis also confirmed this point (Figure 8D). All the above findings suggested that patients in Cluster 2 were more likely to obtain high-risk scores and poor OS and that there was a negative correlation between immune score and risk score. To further investigate the relationship between immune cell infiltration and the risk score, the results revealed that the risk score was negatively related to the functions of CD4 memory activated T cells, follicular helper T cells, regulatory T cells, and CD8 T cells, while positively related with functions of mast cells activated, macrophages M0, and macrophages M2 (Figure 8E). In addition, the correlation between the risk score and the immune checkpoint was also analyzed and demonstrated that the risk score was negatively related to the IDO1, LAG3, PD-1, PD-L1, CTLA4, and TIGIT gene expression (Figure 8F).

## Discussion

As one of the most common tumors of the urinary system, the main treatment for BCa in current clinical practice includes surgical removal of the tumor, such as TURBT, and supplementation with chemotherapy, such as cisplatin. However, with tumor recurrence and drug resistance, BCa patients’ prognosis has not improved within the past several years (Antoni et al., 2017). Recently, immunotherapy as a novel treatment method for multiple cancers has received increasing attention. A clinical trial demonstrated that patients who received atezolizumab had a better prognosis and manageable side effects than patients who received chemotherapy (van der Heijden et al., 2021). However, due to the lack of effective inclusion criteria for the use of immunotherapy, immunotherapy has not become the first choice of clinical first-line treatment.

One of the main reasons why tumors are different from normal tissues is that tumor cells will continue to adapt to the bad environment where they are. Hypoxia is a poor environment that weakens the function of the tumor, while malignant tumor cells are often able to compensate for the process and drive the occurrence of more malignant disease behaviors later (Chae et al., 2016). Therefore, hypoxia as a factor that alters the tumor characteristic microenvironment can accelerate tumor metastasis and enhance drug resistance (Zandberg et al., 2021). Numerous studies have confirmed that there is a hypoxic microenvironment in most solid tumors, including BCa, and the expression of hypoxia-related genes will affect the TME, the efficacy of therapeutic drugs, and patient prognosis (Yang et al., 2017; Liu et al., 2021). However, some previous studies have verified that some HRLs will also affect tumor progression, and the HRL risk model can also predict the patient prognosis well and has an effect on clinical therapy (Shao et al., 2021; Zhang et al., 2021).

In the present study, BCa patients were separated into two molecular subtypes, and the immune microenvironment was evaluated. Univariate Cox regression was used to screen HRLs for subtyping. Four HRLs’ (AC104653, AL136084, AL139393, and LINC00892) low expression levels were used to construct the subtype model. GSEA showed hypoxia genes involved in the MAPK signaling pathway, and the TGFβ signaling pathway was more enriched in the Cluster 2 group than in Cluster 1. TMB analysis showed that different clusters had significantly different mutated genes, such as RB1, KDM6A, and ARID1A, as well as prognosis, which needs further research to recognize its gene functions. Further investigation of the immune microenvironment showed patients in Cluster 2, which had higher immune scores, had higher proportions of CD8 T cells and mast resting cell phenotypes. Moreover, more than 80 differentially expressed immune genes also indicated that hypoxia caused by tumor cell proliferation and malformation of the tumor vascular systems can significantly affect the TME. In addition, based on this subtype, three of them (AC104653, AL136084, and LINC00892) were selected to establish a risk score model by LASSO regression, and patients who obtained higher risk scores predicted bad outcomes compared with the low-risk score. Interestingly, high-risk score patients are sensitive to multiple chemotherapy drugs except for cisplatin compared with low-risk patients. Moreover, nine immune checkpoint analyses also showed that four genes (CTLA4, PD-L2, SIGLEC15, and TIM3) based on these two clusters showed significant difference, and six genes (TIGIT, IDO1, LAG3, PD-1, PD-L1, and CTLA4) have been shown significant differences based on this risk score.

Hypoxia-inducible factor-1 alpha (HIF-1α) has been shown to be activated in the hypoxic microenvironment and to regulate the TGFβ signaling pathway by targeting TGFβ1 (Lazzara et al., 2020). Hypoxia can induce DNA hydroxymethylation and increase the expression of TNFα in the MAPK signaling pathway (Wu et al., 2015). These results are consistent with the results of the GSEA in this study.

Tumor mutational burden is a significant biological marker to indicate the state of tumor mutation and has been described as a powerful tool for identifying candidates associated with tumor immune regulation mechanisms (Klebanov et al., 2019; Cai et al., 2021). Remarkably, in the current study, although the survival analysis illustrated that L-TMB has a worse prognosis than H-TMB, patients in Cluster 2 had a worse survival rate whether in L-TMB or H-TMB compared with Cluster 1, suggesting that patients in Cluster 2 may be most in urgent need of targeted therapies or combination therapies.

The functions of immune cell types are influenced by hypoxia, thereby directly or indirectly inducing tumor development and serving as a barrier to immunotherapy. The expression of HIF-1α in the hypoxic microenvironment promotes cancer glycolysis and evasion of immunosurveillance to promote the function of CD8^+^ T cells (Gropper et al., 2017). In addition, CD4^+^ T cells and NK cells are also regulated to affect anti-tumor immunity and checkpoint molecule expression in the hypoxic microenvironment (Westendorf et al., 2017; Ou et al., 2019). Therefore, these studies have demonstrated that different immune cell subpopulations could respond to hypoxia quite differently, which further confirms the results of this study.

The rise of biological big data mining for patient prognosis has promoted the rapid development of precision medicine in recent years (Mollayeva et al., 2019). Previous studies have performed HRL analyses for multiple types of tumors and built prognostic models, including clear cell renal carcinoma (Zhang et al., 2021). However, there is no further research or model establishment in BCa. Currently, a risk score model is established by LASSO regression, and patients in Cluster 2 obtain higher risk scores than those in Cluster 1. The ROC curve also can illustrate that this kind of risk model in predicting prognosis has more sensitivity and specificity than other clinical features, such as age, gender, tumor grade, or TNM stage. Meanwhile, patients with different risk scores show different outcomes in different clinical groups.

Immune checkpoint inhibitors are a potential treatment for cancer therapy that block key molecules and have shown impressive efficacy against cancer, especially revolutionizing the management of metastatic and locally advanced BCa (de Kouchkovsky et al., 2021; Sun et al., 2021b). In the multifaceted immune regulation of BCa, CTLA4 is one of the primary immune checkpoints. CTLA4 is highly increased in the tumor immune microenvironment, particularly on T regulatory cells (Tregs) upon activation (Arce Vargas et al., 2018). Ipilimumab (IgG1 mAb) is an approved ICI drug of the anti-CTLA4 antibody to be used as a treatment of advanced melanoma and has demonstrated a significant and long-lasting effect in approximately 15 to 20% of treated patients (Schadendorf et al., 2015). PD-L2, similar to PD-L1, is another ligand of PD-1, that has a prominent role in maintaining self-tolerance under normal physiological conditions, limiting T cell activation and proliferation in peripheral tissues (Dowell et al., 2021). Previous studies have demonstrated that multiple tumor patients with PD-L2 expression had worse outcomes, even in the absence of PD-L1, including BCa (Gao et al., 2009; Ariafar et al., 2020). SIGLEC15 is an emerging broad-spectrum target for normalization cancer immunotherapy and is complementary to PD-L1 (Wang et al., 2019). SIGLEC15 can inhibit the CD8^+^ T cell proliferation to promote tumor growth, and the clinical trials of its inhibitors are also ongoing (Hu et al., 2021). TIM3 is expressed on several immune cell types, such as CD4^+^ Th1 cells, CD8^+^ T cells, Tregs, NK cells, and monocytes (Monney et al., 2002; Gorman and Colgan, 2014). However, unlike other immune checkpoints, the ligands for TIM3 have been expressed on a variety of cancer cells (Gorman and Colgan, 2014). In addition, the anti-TIM3 antibodies are being developed (Rotte et al., 2018). TIGIT is expressed in both T cells and NK cells and inhibits immune response mediated *via* triggering CD155 on tumor cells (Anderson et al., 2016; Attalla et al., 2020). LAG3 is expressed on both natural and induced Tregs, T cells, B cells, and DCs, and its receptor ligands include MHC class II molecules expressed on APCs and Galectin-3 (Rotte et al., 2018). IDO1 can inhibit effector T cells and hyper-activating Tregs to induce immune suppression in T cells, while it is not expected to kill tumor cells directly (Jung et al., 2019). However, an early report showed that the combined use of IDO1 inhibitors and PD-1 or PD-L1 inhibitors may enhance the efficacy of checkpoint blockade alone (Jung et al., 2019). In the current study, these nine immune checkpoints had a significant difference in molecular subtype or risk score, suggesting that this molecular subtype and risk score may provide the likelihood of immunotherapy for different BCa patients.

Based on clinical treatment guidelines, cisplatin-based chemotherapy remains the standard therapy in perioperative and first-line metastatic settings (Patel et al., 2020). For patients who have been deemed cisplatin-resistant and harbor tumors with high PD-L1 expression, PD-L1 inhibitor drugs would be used for the first line (Patel et al., 2020). Luckily, according to this study, combining the results of risk scores and molecular subtypes, different patients can choose more sensitive chemotherapy drugs and targeted immunotherapy drugs, single or combined. This is more in line with the concept of individualized treatment in precision medicine.

Although the HRL model has been developed in multiple tumors, this study is the first to develop an HRL model in BCa with some experimental verification. These HRLs were developed based on data from the TCGA database, which contains complete gene expression and clinical and prognosis data of patients with BCa. Meanwhile, the basic experiment in BCa cell lines also verified the expression of HRLs, which is consistent with the result from the TCGA database. However, the current study still has some limitations. First, after searching the GEO database, which contains many gene sequence cohorts, no other available independent lncRNA cohort can be used to validate the usefulness of this model in BCa. Meanwhile, because of the lack of BCa samples, this study cannot verify its effect in clinical practice. In addition, the underlying mechanisms of these four lncRNAs should be investigated by many specific experiments.

## Conclusion

The molecular subtype and risk assessment model based on four HRLs (AC104653, AL136084, AL139393, and LINC00892) can improve the prognostic prediction of BCa patients with different clinical situations. Furthermore, the different expression of ICIs and the different sensitivity of five chemo drugs included in this molecular subtype and risk model might be beneficial targets to explore the development of BCa and design personalized individualized therapy strategies.

## Data Availability Statement

Publicly available datasets were analyzed in this study. This data can be found here: https://portal.gdc.cancer.gov/, http://www.gsea-msigdb.org/gsea/.

## Author Contributions

XJ and XC constructed this study. XC and YZ collected and analyzed the data and drafted the manuscript. FW, XZ, and QF made the figures. XY, JL, and XC revised the manuscript. All authors read and approved the final manuscript.

## Conflict of Interest

The authors declare that the research was conducted in the absence of any commercial or financial relationships that could be construed as a potential conflict of interest.

## Publisher’s Note

All claims expressed in this article are solely those of the authors and do not necessarily represent those of their affiliated organizations, or those of the publisher, the editors and the reviewers. Any product that may be evaluated in this article, or claim that may be made by its manufacturer, is not guaranteed or endorsed by the publisher.

## Footnotes

1
https://portal.gdc.cancer.gov/

2
http://www.gsea-msigdb.org/gsea/

3
https://www.cancerrxgene.org/

## Supplementary Material

Supplementary Figure 1 Identification of hypoxia genes and lncRNAs. The heatmap **(A)** and a volcano plot **(B)** of the different expressions of hypoxia genes between normal and BCa samples. **(C)** The protein-protein interaction (PPI) network of 15 hub hypoxia genes. **(D)** The mRNA-lncRNA network of hypoxia genes and HRLs.

Supplementary Figure 2 The heatmap of the consensus clustering of HRLs.

Supplementary Figure 3 GSEA for the different molecular subtypes of HRLs.

Supplementary Figure 4 The heatmap of the expression of different molecular subtypes.

Supplementary Figure 5 The boxplot of the expression of 9 immune checkpoints in normal and BCa samples.

Supplementary Figure 6 The Kaplan-Meier curve of OS of the risk model.

Supplementary Figure 7 The Survival-dependent ROC curves of risk model prognostic indicators.

Supplementary Figure 8 The boxplot of the clinicopathological significance of the risk model.
