# Supplementary figures and images for: A Novel Assessment Model Based on Molecular Subtypes of Hypoxia-Related LncRNAs for Prognosis of Bladder Cancer
Source: Front Cell Dev Biol. 2021 Nov 15;9:718991. doi: 10.3389/fcell.2021.718991 (PMC8634255; doi:10.3389/fcell.2021.718991)

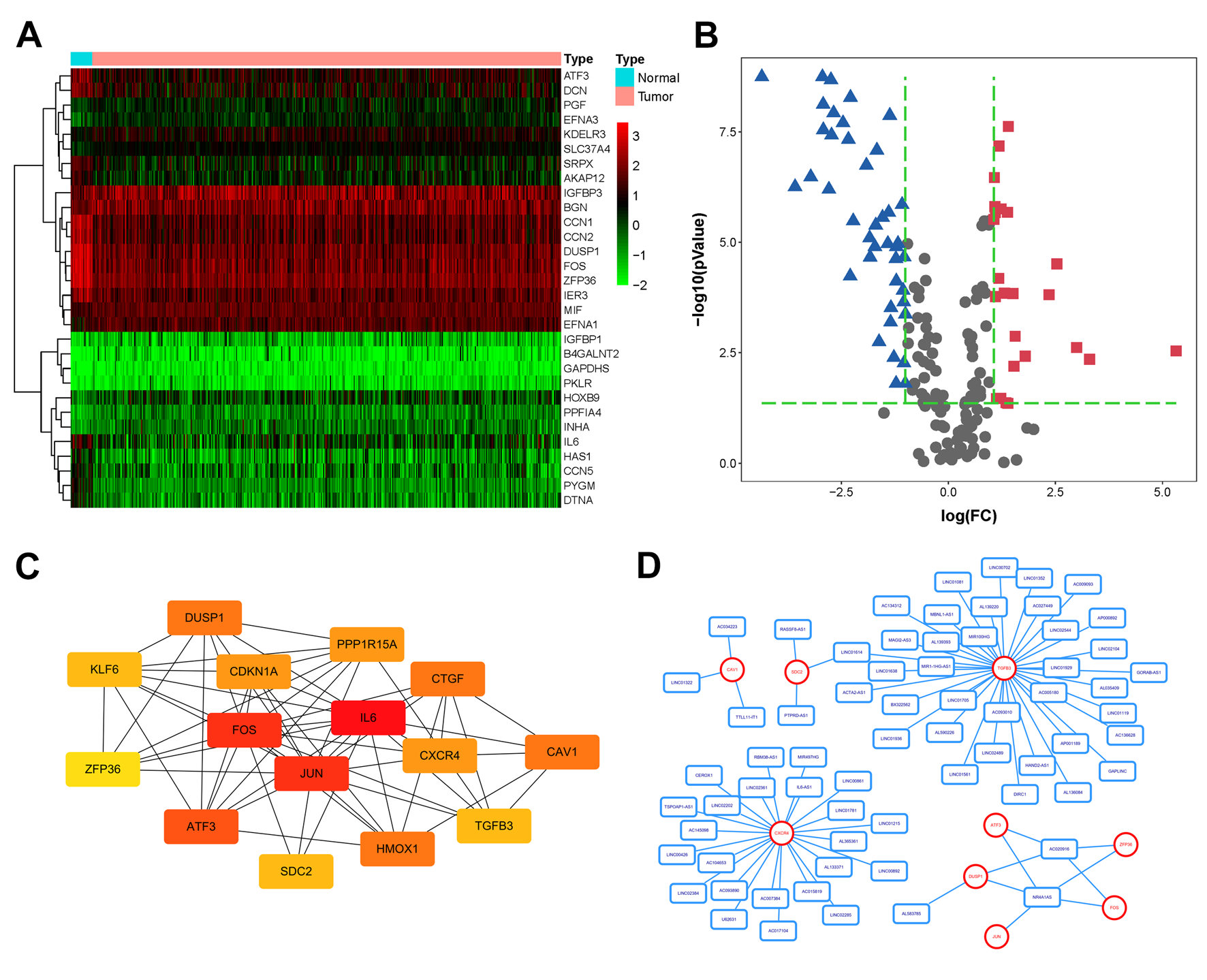

Supplement: Supplementary Figure 1 — Identification of hypoxia genes and lncRNAs. The heatmap (A) and a volcano plot (B) of the different expressions of hypoxia genes between normal and BCa samples. (C) The protein-protein interaction (PPI) network of 15 hub hypoxia genes. (D) The mRNA-lncRNA network of hypoxia genes and HRLs. [file Image_1.TIF]

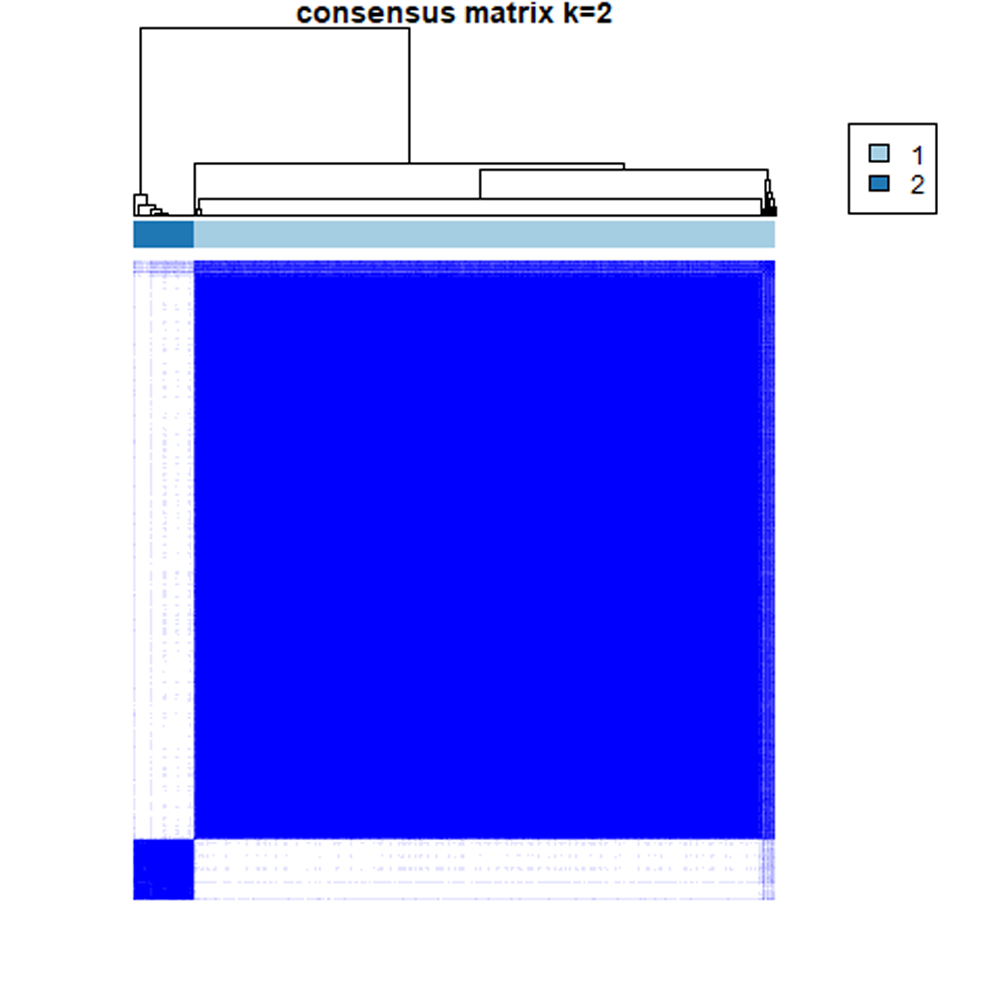

Supplement: Supplementary Figure 2 — The heatmap of the consensus clustering of HRLs. [file Image_2.TIF]

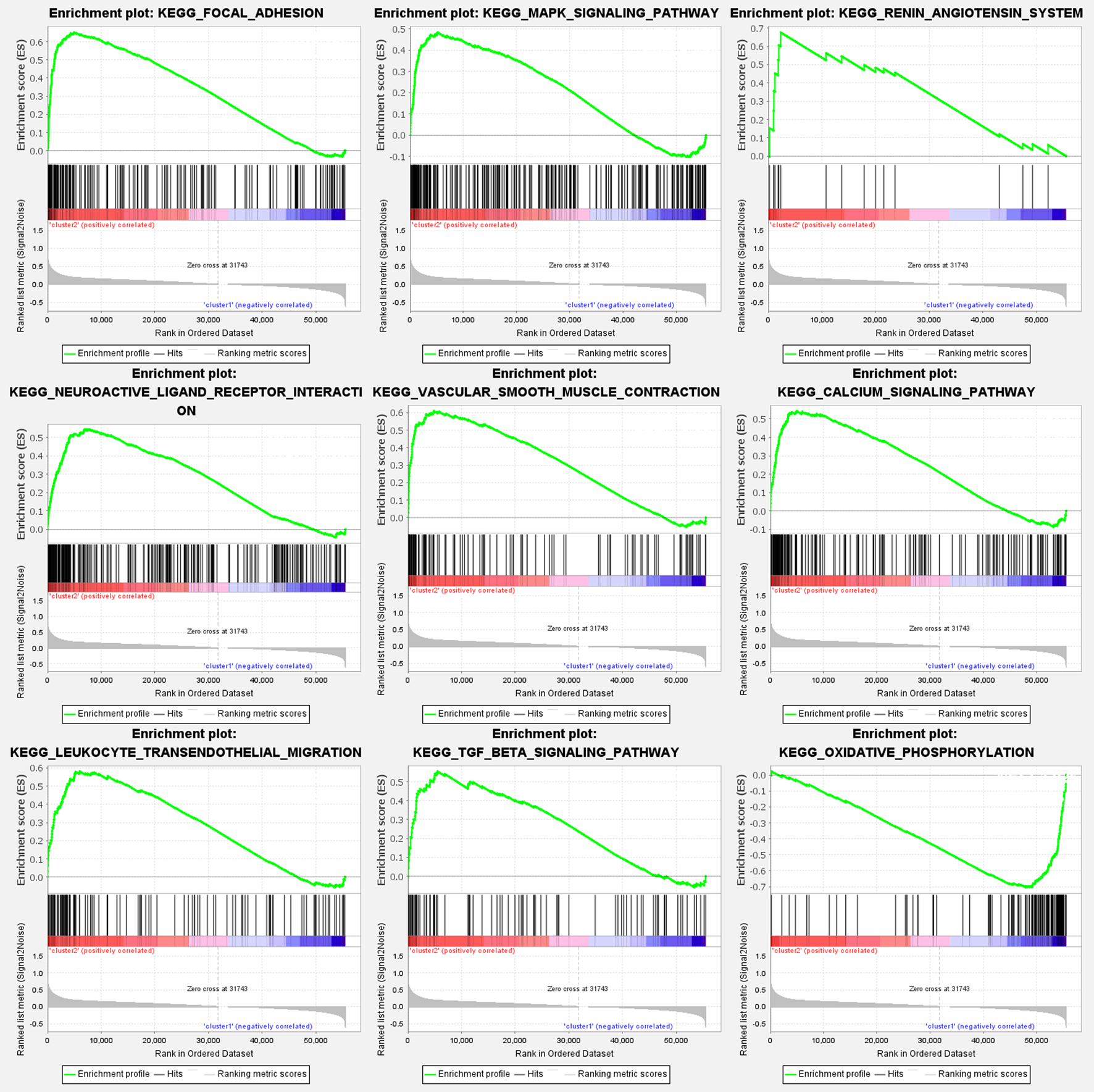

Supplement: Supplementary Figure 3 — GSEA for the different molecular subtypes of HRLs. [file Image_3.TIF]

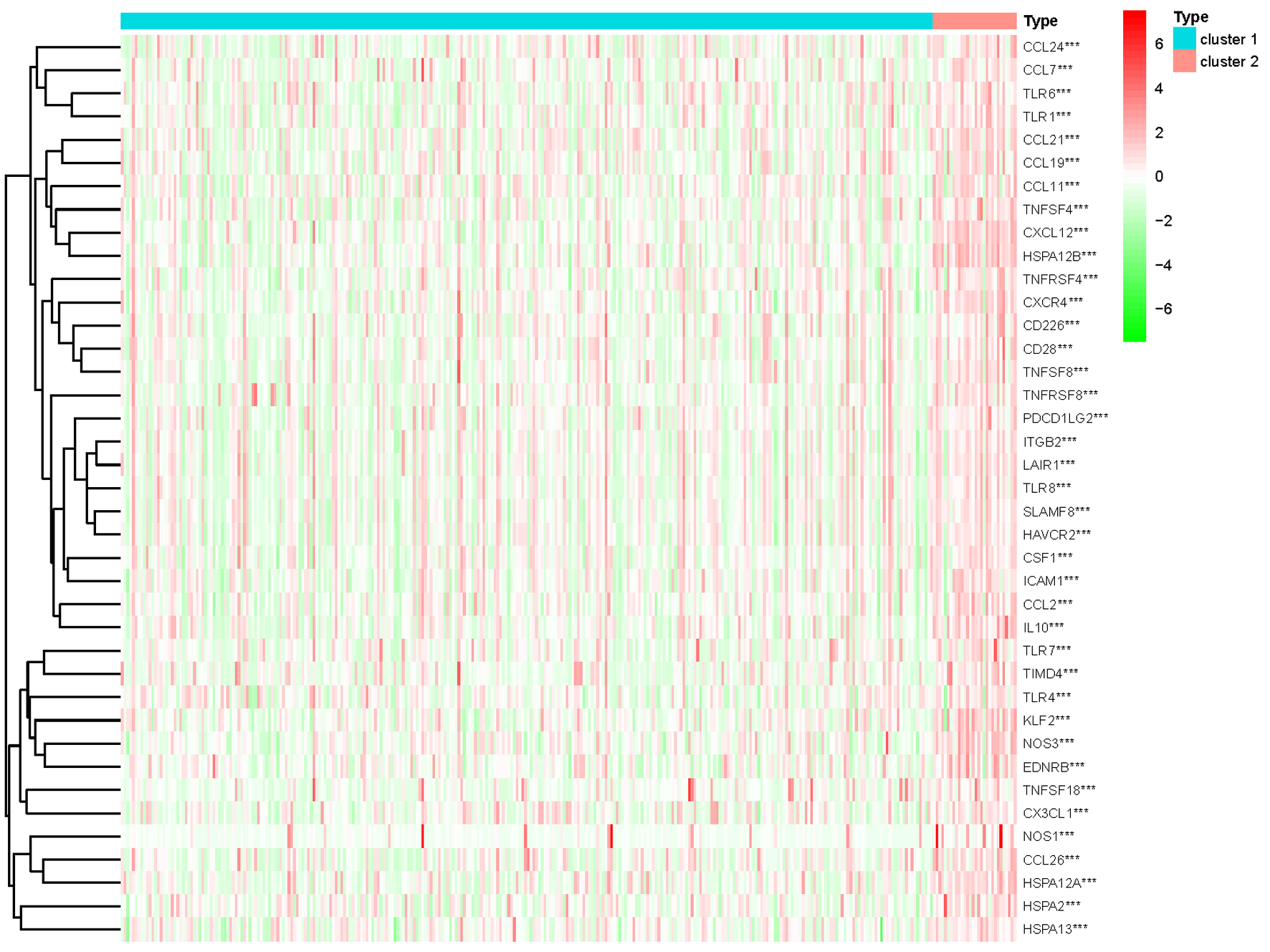

Supplement: Supplementary Figure 4 — The heatmap of the expression of different molecular subtypes. [file Image_4.TIFF]

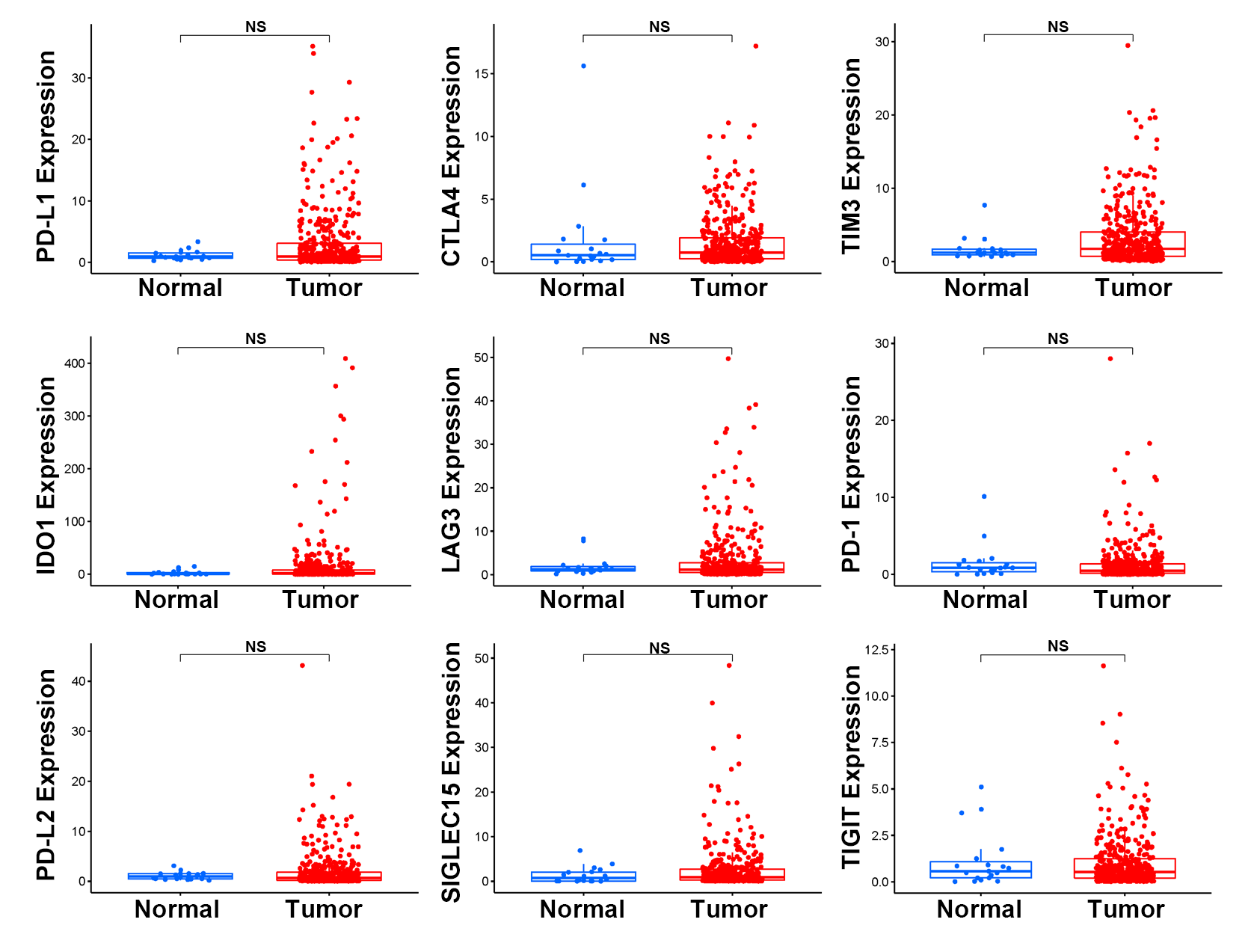

Supplement: Supplementary Figure 5 — The boxplot of the expression of 9 immune checkpoints in normal and BCa samples. [file Image_5.TIF]

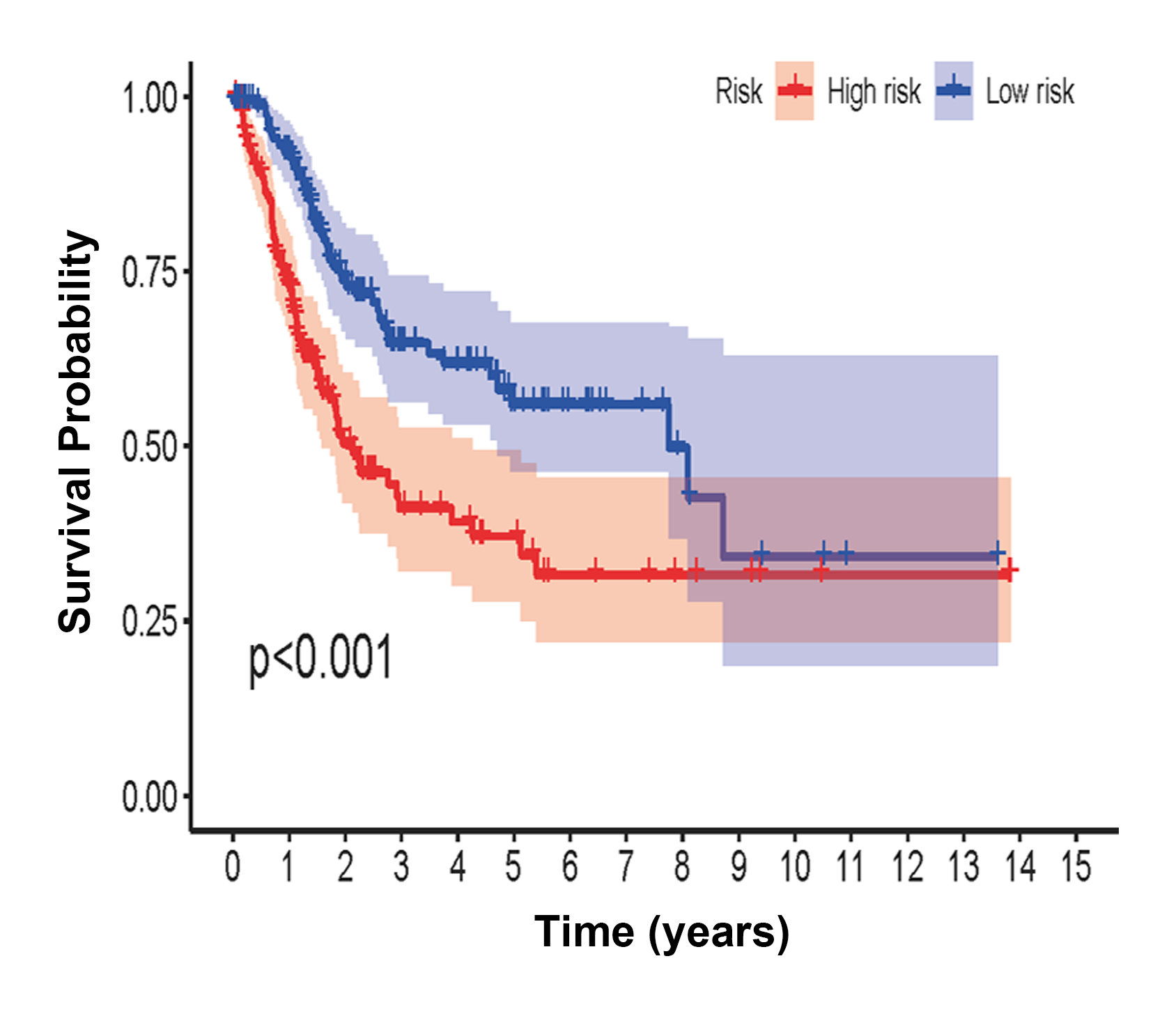

Supplement: Supplementary Figure 6 — The Kaplan-Meier curve of OS of the risk model. [file Image_6.TIF]

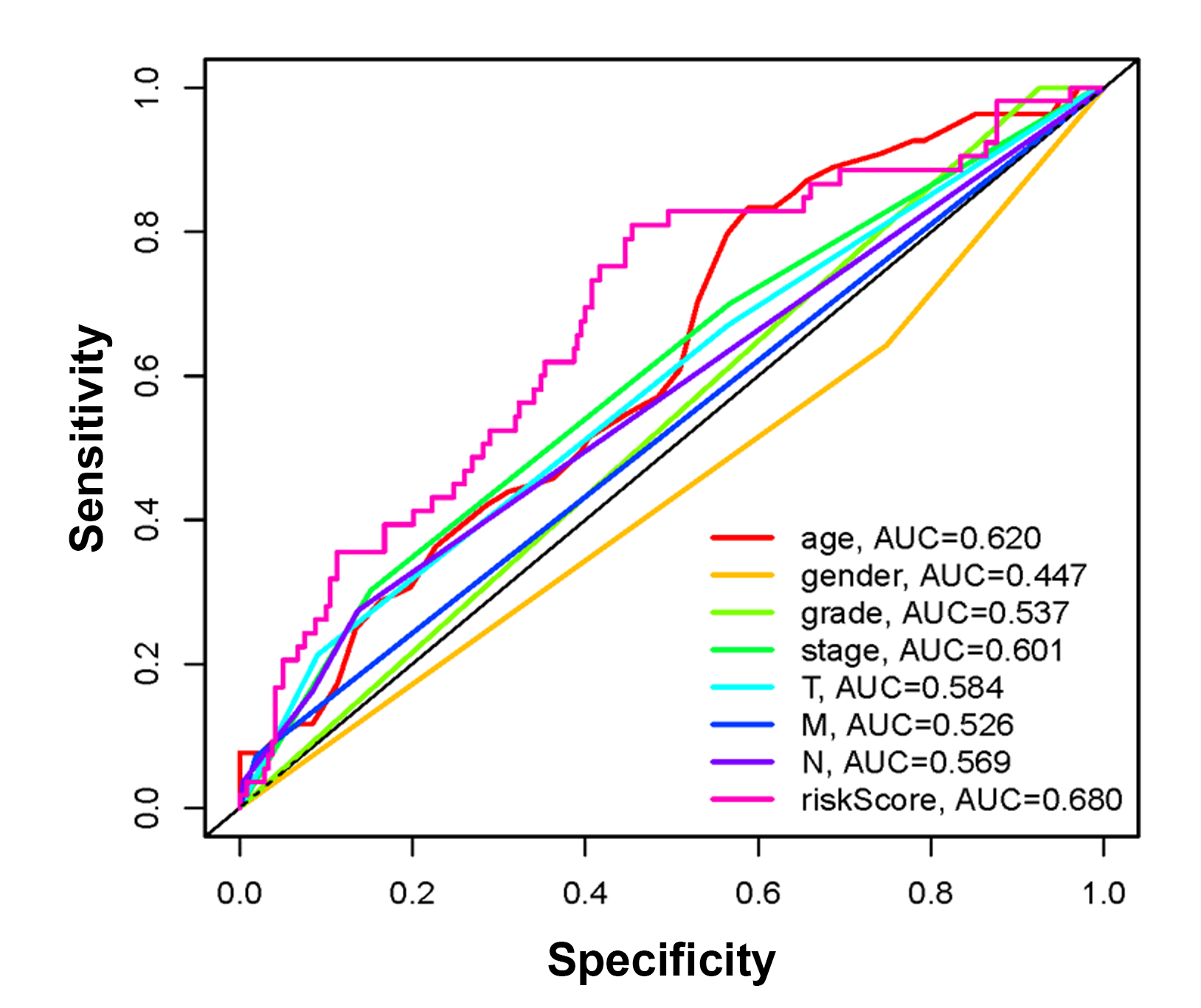

Supplement: Supplementary Figure 7 — The Survival-dependent ROC curves of risk model prognostic indicators. [file Image_7.TIF]

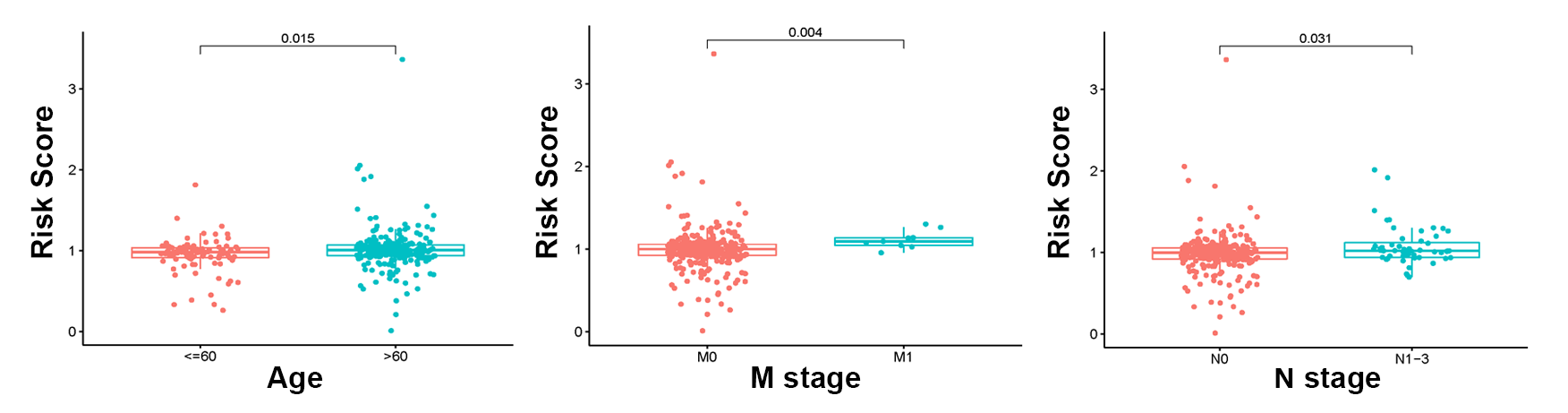

Supplement: Supplementary Figure 8 — The boxplot of the clinicopathological significance of the risk model. [file Image_8.TIF]
