# Supplementary material for: A Novel Assessment Model Based on Molecular Subtypes of Hypoxia-Related LncRNAs for Prognosis of Bladder Cancer
Source: Front Cell Dev Biol. 2021 Nov 15;9:718991. doi: 10.3389/fcell.2021.718991 (PMC8634255; doi:10.3389/fcell.2021.718991)
Supplement: Supplementary file 9 [file Table_1.DOCX]

**Supplementary Table S1. Primer information**

| **Primer Symbol** | **Primer direction** | **Sequences (5’ to 3’)** |
| --- | --- | --- |
| AC104653 | Forward | CGGACGGCTGGCTATCTTAC |
|  | Reverse | TTTCCCAGTTTTGGTGCTGC |
| AL136084 | Forward | ATGACCAGACACATCACACCC |
|  | Reverse | TCTTTCTCCCAACCAGAAGCC |
| AL139393 | Forward | AGCTGGGCTTTTCTCCTTCG |
|  | Reverse | TCCTGGCCTACGTGAGTAAAT |
| LINC00892 | Forward | TGGATGTTCTTTGCTGGGCT |
|  | Reverse | GCTCGTTCTTCTCTTACGGCT |
| GAPDH | Forward | GTCAAGGCTGAGAACGGGAA |
|  | Reverse | AAATGAGCCCCAGCCTTCTC |
